# Doxorubicin-loaded iron oxide nanoparticles for glioblastoma therapy: a combinational approach for enhanced delivery of nanoparticles

**DOI:** 10.1038/s41598-020-68017-y

**Published:** 2020-07-09

**Authors:** Mohammad Norouzi, Vinith Yathindranath, James A. Thliveris, Brian M. Kopec, Teruna J. Siahaan, Donald W. Miller

**Affiliations:** 1 Department of Biomedical Engineering, University of Manitoba, Winnipeg, MB Canada; 2 Department of Pharmacology and Therapeutics, University of Manitoba, A205 Chown Bldg., 753 McDermot Avenue, Winnipeg, MB Canada; 3 Department of Human Anatomy and Cell Science, University of Manitoba, Winnipeg, MB Canada; 4 Department of Pharmaceutical Chemistry, University of Kansas, Lawrence, KS USA

**Keywords:** Nanobiotechnology, Nanoscale materials

## Abstract

Although doxorubicin (DOX) is an effective anti-cancer drug with cytotoxicity in a variety of different tumors, its effectiveness in treating glioblastoma multiforme (GBM) is constrained by insufficient penetration across the blood–brain barrier (BBB). In this study, biocompatible magnetic iron oxide nanoparticles (IONPs) stabilized with trimethoxysilylpropyl-ethylenediamine triacetic acid (EDT) were developed as a carrier of DOX for GBM chemotherapy. The DOX-loaded EDT-IONPs (DOX-EDT-IONPs) released DOX within 4 days with the capability of an accelerated release in acidic microenvironments. The DOX-loaded EDT-IONPs (DOX-EDT-IONPs) demonstrated an efficient uptake in mouse brain-derived microvessel endothelial, bEnd.3, Madin–Darby canine kidney transfected with multi-drug resistant protein 1 (MDCK-MDR1), and human U251 GBM cells. The DOX-EDT-IONPs could augment DOX’s uptake in U251 cells by 2.8-fold and significantly inhibited U251 cell proliferation. Moreover, the DOX-EDT-IONPs were found to be effective in apoptotic-induced GBM cell death (over 90%) within 48 h of treatment. Gene expression studies revealed a significant downregulation of TOP II and Ku70, crucial enzymes for DNA repair and replication, as well as MiR-155 oncogene, concomitant with an upregulation of caspase 3 and tumor suppressors i.e., p53, MEG3 and GAS5, in U251 cells upon treatment with DOX-EDT-IONPs. An in vitro MDCK-MDR1-GBM co-culture model was used to assess the BBB permeability and anti-tumor activity of the DOX-EDT-IONPs and DOX treatments. While DOX-EDT-IONP showed improved permeability of DOX across MDCK-MDR1 monolayers compared to DOX alone, cytotoxicity in U251 cells was similar in both treatment groups. Using a cadherin binding peptide (ADTC5) to transiently open tight junctions, in combination with an external magnetic field, significantly enhanced both DOX-EDT-IONP permeability and cytotoxicity in the MDCK-MDR1-GBM co-culture model. Therefore, the combination of magnetic enhanced convective diffusion and the cadherin binding peptide for transiently opening the BBB tight junctions are expected to enhance the efficacy of GBM chemotherapy using the DOX-EDT-IONPs. In general, the developed approach enables the chemotherapeutic to overcome both BBB and multidrug resistance (MDR) glioma cells while providing site-specific magnetic targeting.

## Introduction

Glioblastoma multiforme (GBM) is the most common and aggressive form of malignant gliomas whose current standard of care involves surgical recession followed by chemotherapy and radiotherapy^1,2^. Nevertheless, the median survival of GBM patients who receive the current standard of care is 14.6 months post-diagnosis, and 5-year survival rate is only 9.8%^3^. The extensive infiltration of GBM tumors in addition to the presence of the blood–brain barrier (BBB) limits chemotherapeutic options. The BBB is made of tight junctions between endothelial cells and surrounding astrocyte foot processes, controlling the passage of substances from the bloodstream into the brain^4,5^. Besides the tight junctions that restrict the paracellular passage of drugs, brain endothelial cells also express a number of efflux transporters such as P-glycoprotein (P-gp) and breast cancer resistance protein (BCRP), thus limiting drug penetration into the brain^6^. In this respect, a majority of the current chemotherapeutics available to treat GBM have BBB liabilities that negatively impact on therapeutic efficacy^7^. As a result, the chemotherapeutic options are limited and those drugs that are used often require high doses that pose severe systemic toxicity to the normal tissues^8,9^.

To address these issues, numerous engineered nanoparticles (e.g. iron oxide nanoparticles, gold nanoparticles, nanoliposomes) have been used as drug delivery systems capable of penetrating the BBB and delivering therapeutic agents to the GBM tumor site^10^. Iron oxide nanoparticles [IONPs, magnetite (Fe_3_O_4_) or maghemite (γ-Fe_2_O_3_)], *inter alia*, have found extensive applications in cancer theranostics by virtue of their tunable size-dependent magnetic properties. The IONPs are biocompatible and biodegradable, and can be incorporated into the body’s iron cycle upon degradation^5,11^. Furthermore, the surface of IONPs can be further modified in order to (i) improve their biocompatibility and aqueous dispersibility, (ii) prolong their circulating time in blood through minimizing nonspecific phagocytosis by the reticuloendothelial system (RES) and also, (iii) provide active sites for drug loading^10,12^. To this end, a variety of biopolymers such as poly(ethylene glycol) (PEG)^13^, poly(ethylene imine) (PEI)^14^, dextran^15^ and chitosan^16^ have been employed for surface modification of IONPs.

Generally, the inherent magnetic properties of the IONPs make them a promising option for both magnetic resonance imaging (MRI) as a contrast agent, and for site-specific magnetic targeting using an external magnetic field^11^. Several types of IONPs have been developed as MRI contrast agents in clinical trials such as Ferumoxide (Feridex), Ferumoxytol (Feraheme), Ferucarbotran (Resovist) and ferumoxtran-10 (Combidex), due to their effective reduction of T1, T2, and T2* relaxation times^10^. Moreover, several IONPs have been fabricated as an efficacious nanocarrier for anti-cancer drugs such as DOX^17^, paclitaxel^18^ and 5-fluorouracil^19^, albeit none of these have progressed to clinical trials yet.

Doxorubicin (DOX) is an anthracycline with potent antitumor activity in a variety of cancer cells^20^. Generally, DOX intercalates base pairs of the DNA strands, thus inhibiting the synthesis of DNA as well as RNA through blocking the replication and transcription processes. In addition, DOX inhibits topoisomerase II (TOP2), an enzyme regulating DNA under- and over-winding, further preventing DNA replication, transcription and repair. Generation of free radicals is another mechanism of DOX activity that induces oxidative damage resulting in cleavage or degradation of DNA^20,21^. DOX is considered as one of the most effective chemotherapeutics and is currently indicated by the FDA for a variety of neoplastic conditions such as leukemia, neuroblastoma, soft tissue and bone sarcoma, breast carcinoma, ovarian carcinoma, bladder carcinoma, thyroid carcinoma, gastric carcinoma, Hodgkin's disease, malignant lymphoma and bronchogenic carcinoma^20^. Intravenous (i.v.) administration of DOX, however, exhibits several adverse effects including dose-limiting cardiotoxicity and myelosuppression^20^. The underlying mechanisms of cardiotoxicity are mainly attributed to the overproduction of reactive oxygen species (ROS) and inhibition of topoisomerase IIβ (Top2β)^22^. While DOX is a potent and effective chemotherapeutic *in vitro* against cell lines derived from malignant gliomas (IC50 of DOX is 0.5 µM vs temozolomide, the standard agent in glioma chemotherapy, that has an IC50 of 35 µM on U251 GBM cell line)^23,24^, its inadequate penetration across the BBB severely constrains its effective use in treating GBM patients. However, the therapeutic efficacy of either pegylated liposomal DOX^25^ or its intratumoral administration^26^ in patients with malignant gliomas has been reported.

Taken together, development of drug delivery systems for DOX with a capability of site-specific drug release and improved BBB penetration would represent a significant improvement for treatment of GBM. Thus far, several nanotechnology-based DOX formulations have been developed. Doxil is a pegylated liposomal formulation of doxorubicin approved by the FDA for administration in a variety of human cancers^27^. In addition, other nanotechnology-based DOX formulations such as NK-911 (DOX-conjugated poly-aspartic acid/polyethylene glycol micelles, phase II, metastatic pancreatic cancer) and Livatag (DOX-loaded polyalkylcyanoacrylate nanoparticles, phase III, primary liver cancer) are under clinical trials^20^.

In this study, EDT-coated IONPs were developed as a delivery system for DOX and the anti-cancer effects of the formulation were investigated in vitro on GBM cells. EDT is a biocompatible coating that provides many negative charged sites on the surface of the nanoparticles^28,29^ that can be utilized for ionic interaction with positively charged DOX molecules. Previous studies have demonstrated the biocompatibility of the EDT-IONPs in healthy Balb/c mice and the ability of transient opening of BBB to increase the brain penetration of these nanoparticles^29^. In this study, drug-loaded EDT-IONP together with a cadherin binding peptide to transiently enhance the permeability of IONPs was shown to be effective in a BBB-GBM co-culture model. This combinational approach of using a cadherin binding peptide and an external magnetic field together not only enhanced the penetration of the nanoparticles but also resulted in increased therapeutic response and apoptosis in GBM cells.

## Results and discussion

### Characterization of EDT-IONPs

**Figure 1:**
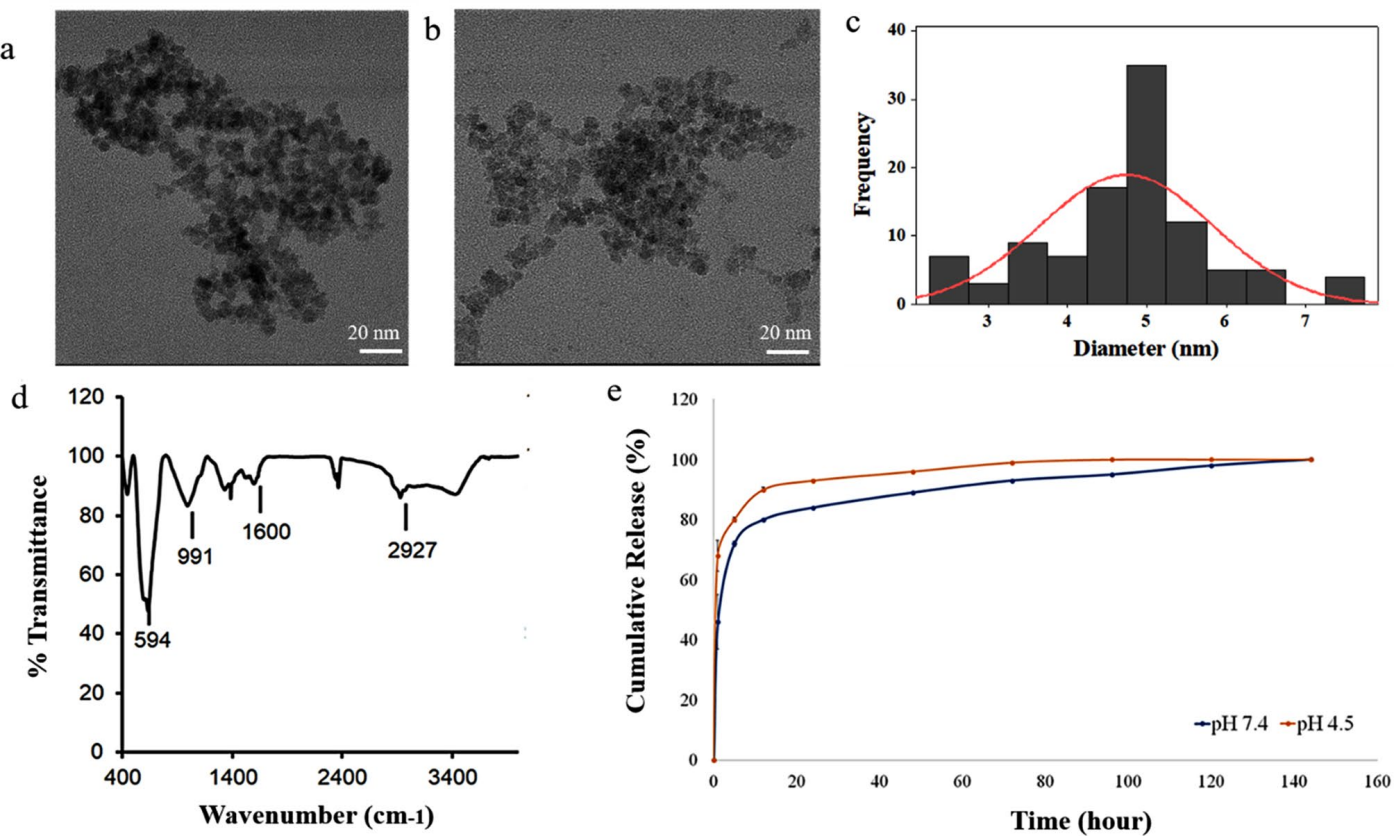
Characterization of nanoparticles: TEM images of (**a**) EDT-IONPs, and (**b**) DOX-EDT-IONPs; (**c**) histogram of EDT-IONP size distribution from the measurement of 100 particles; (**d**) FTIR spectrum of EDT-IONP, (**e**) release of DOX from the DOX-EDT-IONPs in pH 7.4 and 4.5.

**Figure 2:**
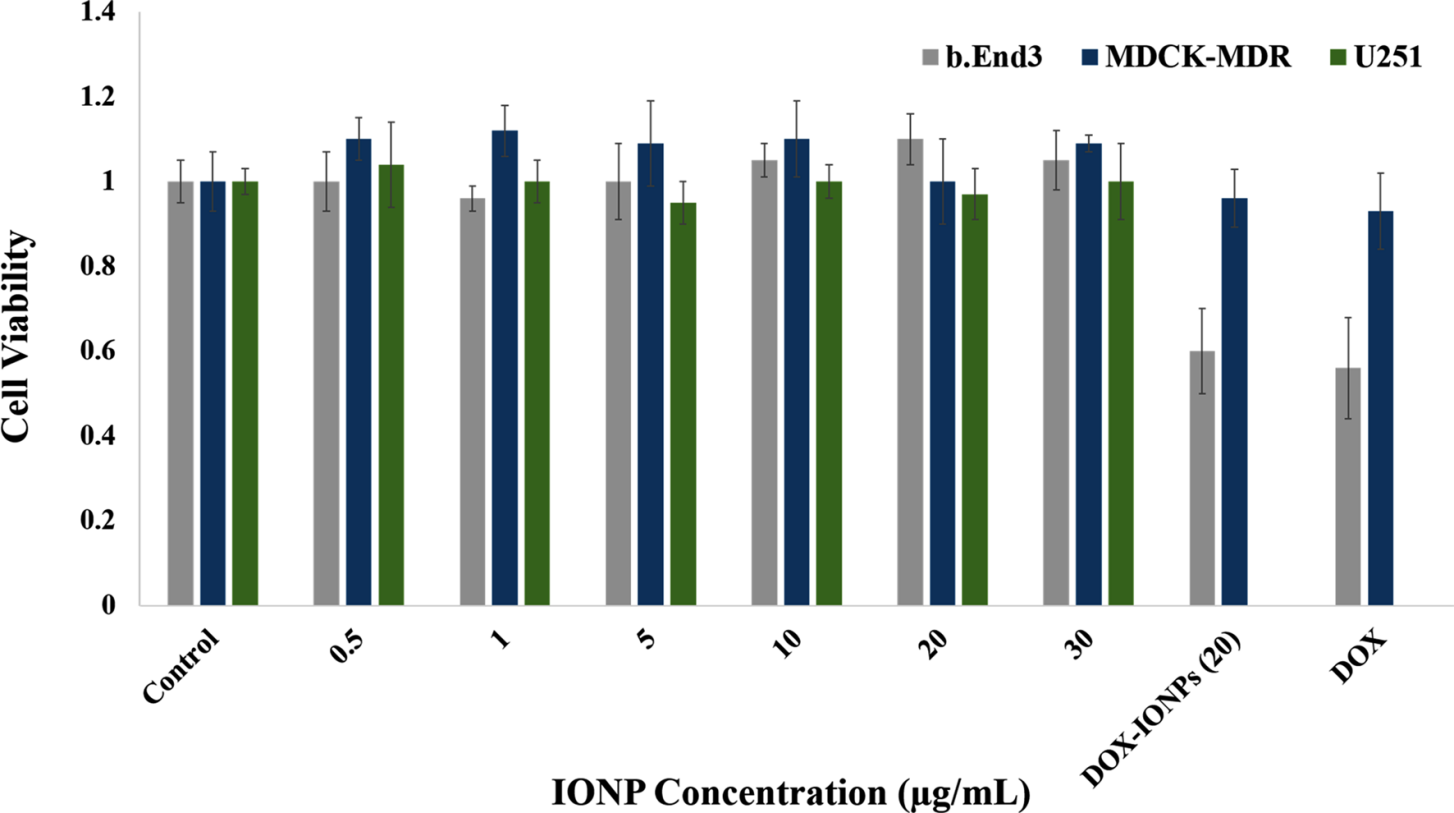
Biocompatibility of EDT-IONPs on b.End3, MDCK-MDR1 and U251 cell lines after 48-h treatment using MTT assay (n = 5). DOX concentration was 1 µg/mL. The Y-axis represents cell viability compared to the control.

**Figure 3:**
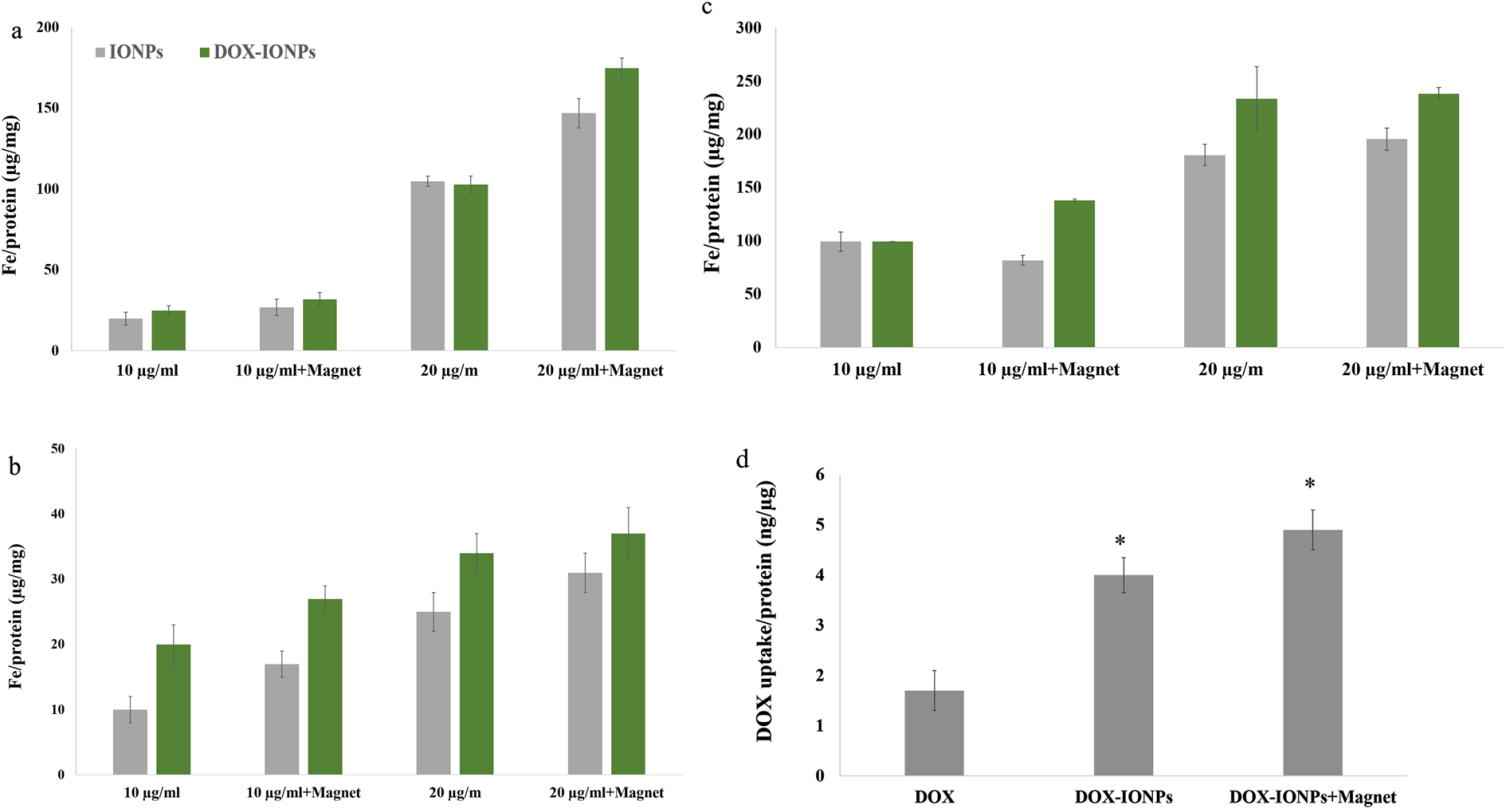
Uptake of EDT-IONPs and DOX-EDT-IONPs by (**a**) b.End 3, (**b**) MDCK-MDR1, and (**c**) U251 after 4-h treatment. (**d**) Uptake of DOX by U251 cells treated with either DOX, DOX-EDT-IONPs, or DOX-EDT-IONPs + Magnet after 2 h. *Indicates a significant difference compared to DOX at p < 0.05. Data is presented as mean ± S.D, and n = 3 (three replications). IONPs and DOX-IONPs represent for EDT-IONPs and DOX-EDT-IONPs.

The TEM image illustrates EDT-IONPs (Fig. 1a) and DOX-EDT-IONPs (Fig. 1b) with a quasi-spherical morphology and a core size of 4.76 ± 0.7 nm (Fig. 1c). The hydrodynamic diameter (D_H_) and zeta potential (ζ) of the EDT-IONPs were 51.8 ± 1.3 nm, and − 27.3 ± 1.0 mV, respectively. The suspensions of both nanoparticles were stable at physiological pH (Fig. 1S). The FTIR spectrum of the EDT-IONPs is shown in Fig. 1d. The Fe–O–Fe stretching of the core was observed at 594 cm^−1^ and the Si–O–Si stretching band of the aminosilane shell was found at 991 cm^−1^. The carbonyl stretching band of EDT coating and the C-H stretching (of propyl group) bands were observed at 1,600 cm^−1^ and 2,927 cm^−1^, respectively. The energy-dispersive X-ray spectrum of EDT-IONPs for elemental analysis was also reported in supplementary materials (Fig. 2S). The powder X-ray diffraction pattern of the nanoparticles was also shown in Fig. 3S, whose peaks were indexed to cubic unit cell characteristic of magnetite/maghemite (Fe_3_O_4_/$\gamma -$Fe_2_O_3_) phase as previously reported^30^. The magnetic properties and additional physicochemical characterizations of the IONPs were previously reported^30,31^.

### Characterization of DOX-IONPs

The DOX loading efficiency on the EDT-IONPs was calculated to be 5 ± 0.05%. The DOX-EDT-IONPs had a ζ of 0.0 ± 0.02 mV compared to − 20.05 ± 2.7 mV for EDT-IONPs. The change in surface charge of the nanoparticles upon drug loading can be attributed to the electrostatic interactions between the amine groups of DOX and carboxylic acid groups of EDT coating. In addition, the D_H_ of the EDT-IONPs increased from 51.8 ± 1.3 nm (polydispersity index: PDI 0.14) to 75.5 ± 3.2 nm (PDI 0.27) upon DOX loading. The release profile of DOX from the nanoparticles is depicted in Fig. 1e. The nanoparticles demonstrated a burst release of 42 ± 5% within the initial 3 hours, while the remaining coated DOX gradually released within a 4-day period. Moreover, upon release of the loaded-DOX from nanoparticles within 4 days, the surface charge of the nanoparticles became negative again and returned to − 25.69±2.8 mV. Release studies performed at pH 4.5 also showed an accelerated initial release of DOX from the nanoparticles with up to 64 ± 4% within the initial hours. The enhanced release at pH 4.5 was due to the reduced electrostatic interactions between DOX and IONPs^10^. The increased release of DOX observed under these acidic conditions is similar to previous reports with DOX-IONP in the acidic tumor microenvironment or acidic cellular compartments such as endosomes^32,33^.

The release of DOX from the IONPs observed in the present study was similar to previous reports with polymer-based nanoparticles. Poly-L-arginine/chitosan-coated iron oxide nanoparticles exhibited 40% and 65% release of DOX within 2 h at pHs 7 and 5, respectively^34^. Although covalent bonding of DOX to the surface of the nanoparticles can result in increased loading and reduced initial burst release, these advantages are countered by potential reductions in the total release of the drug from the nanoparticles. For instance, when DOX was covalently conjugated to iron oxide nanoparticles via a pH-sensitive hydrazone linkage, there was a 29% burst release within 2 h. However, only 4% of the loaded DOX was further released within 24 h and the cumulative release was only around 35% under acidic pH conditions^35^. The release rate observed in the present study is well-suited for the proposed delivery approach involving transient opening of the BBB. Previous *in vivo* studies using the cadherin peptides for transient opening of the BBB indicated a therapeutic delivery window of approximately 60 minutes following treatment^36^. Thus, the DOX-EDT-IONPs would be expected to enter the brain within an hour, while carrying over 60% of the initial concentration of the loaded DOX. Moreover, the rapid release of DOX (within an hour) from the DOX-EDT-IONPs that magnetically has been drawn to the target site, can increase the chance of DOX entering the brain through the transiently open tight junctions of the BBB to provide a higher concentration of the drug within the brain.

### Biocompatibility of EDT-IONPs

Biocompatibility of the EDT-IONPs on U251, bEnd.3 and MDCK-MDR1 cells was studied. No cytotoxicity was observed in any of these three cell lines following treatment with EDT-IONPs at concentrations ranging from 0.25 to 30 µg/mL (Fig. 2). Furthermore, treatment of cells with either DOX-EDT-IONPs or free DOX (1 µg/mL) resulted in an approximately 40% reduction in the bEnd.3 cell viability, while no significant cytotoxicity was observed in MDCK-MDR1. This is likely attributed to the presence of P-gp efflux transporter that restricts uptake of DOX by MDCK-MDR1 cells^37^.

In order to deliver an effective concentration of both DOX and EDT-IONPs to GBM cells in our studies, the concentration of 20 µg/mL of EDT-IONPs was selected for use in the remaining studies. This concentration, which was well tolerated in various cell lines, enables delivering enough DOX to observe cytotoxicity on the tumor cells. We previously reported biocompatibility of IONPs on endothelial, astrocyte and neuron cells at a concentration up to 100 µg/mL^38^. Moreover, iron oxide nanoparticles clinically demonstrate acceptable biocompatibility and they are captured by the reticuloendothelial system (RES), by which the iron is incorporated into the body’s iron cycle^5^. In practice, iron oxide nanoparticles are coated with biocompatible and hydrophilic materials, diminishing the non-specific protein adsorption on the nanoparticle surface and decreasing their recognition and clearance by the RES, thereby their circulation time, as well as accumulation in the brain tumor can be augmented^39^.

### Cellular uptake of the EDT-IONPs and DOX

**Figure 4:**
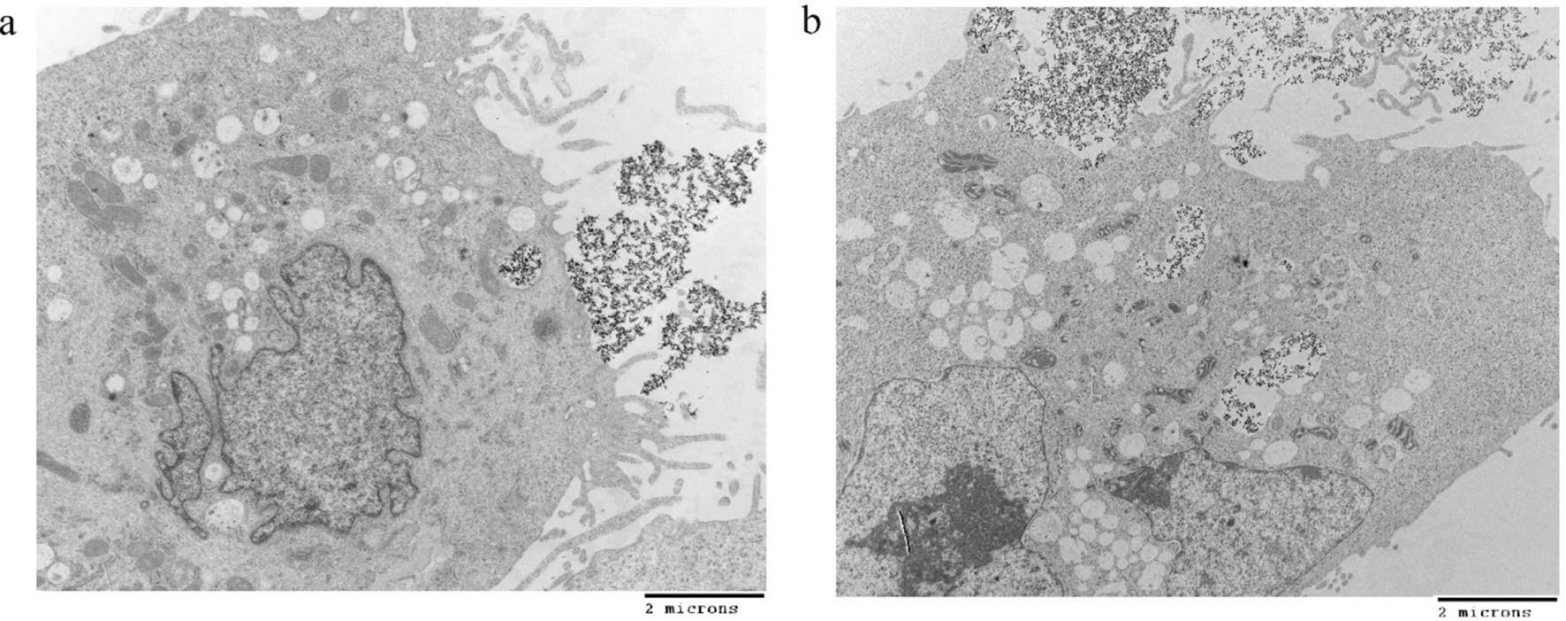
TEM images of nanoparticles uptake by U251 cells cell after 4 h of the treatment, (**a**) EDT-IONPs and (**b**) DOX-EDT-IONPs.

The cellular uptakes of EDT-IONPs and DOX-EDT-IONPs in bEnd.3 (Fig. 3a), MDCK-MDR1 (Fig. 3b), and U251 (Fig. 3c) were evaluated. The uptake of both EDT-IONP and DOX-EDT-IONP was concentration-dependent, with the drug-loaded IONPs generally displaying greater cellular uptake than that of the EDT-IONPs (Fig. 3). Moreover, the cellular uptake of the nanoparticles was augmented in the presence of a magnetic field. The bEnd.3 and MDCK-MDR1 demonstrated a greater uptake of the nanoparticles than that of U251. While mechanistically, there are many types of endocytosis pathways present in endothelial and epithelial cells^40,41^, our previous studies suggest that caveolin- dependent endocytosis is likely the major contributor to EDT-IONPs internalization in the bEnd.3 and MDCK cells^42^. The TEM images taken from U251 cells following EDT-IONP or DOX-EDT-IONP (Fig. 4) showing nanoparticle sequestration in intracellular vesicles (i.e., endosomes/lysosomes) is also supportive of an endocytosis pathway for uptake of the nanoparticles. Regarding the higher uptake of DOX-EDT-IONPs compared to EDT-IONPs observed in the MDCK-MDR1 and U251 cells, the neutral surface charge of the former would favour the cellular uptake of the drug-loaded nanoparticles. Previous studies with IONPs examining the role of surface charge and cellular uptake have reported greater uptake of positively charged IONPs compared to negatively charged IONPs^38^.

In terms of drug accumulation in U251 GBM cells, treatment with DOX-EDT-IONPs, was more effective than DOX alone. In the present study, the DOX-EDT-IONP resulted in approximately 2-fold greater uptake compared to an equal concentration of DOX in solution (Fig. 3d). In addition, application of an external magnetic field further enhanced the DOX accumulation in the U251 cells (2.8 ± 0.5-fold, Fig. 3c). In practice, the efficacy of chemotherapy with DOX is limited by the multiple drug resistance (MDR) mechanisms due to the overexpression of ATP-binding cassette (ABC) and P-gp efflux transporter in cancer cells. The expression of P-gp in U251 has been previously reported^43^. In this regard, Wang et al.,^43^ reported that co-administration of β-asarone and TMZ could decrease P-gp and MDR1 expression in U251, thus promoting TMZ’s entry into the GBM cells. Therefore, in this study, the DOX loaded on the nanoparticles could bypass the P-gp efflux system, leading to higher DOX’s uptake in the GBM cells^5,35^. Similarly, higher uptake of DOX upon treatment of C6 glioma cells with DOX-loaded-polysorbate 80-SPIONs in comparison to that of free DOX was reported through endocytosis of the nanoparticles^10^.

### Cytotoxicity of DOX-EDT-IONPs on cancer cell

**Figure 5:**
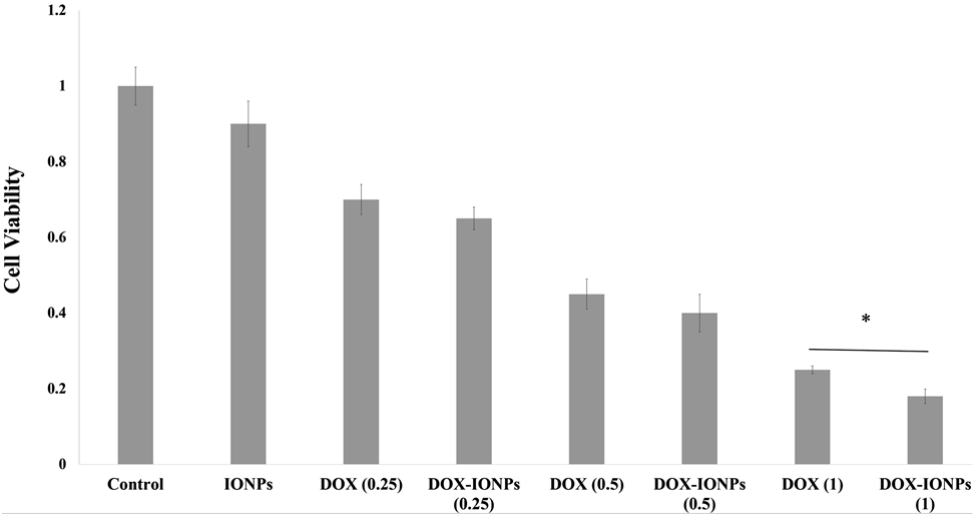
Cytotoxicity assessment of various concentrations of DOX and DOX-EDT-IONPs (0.25, 0.5 and 0.1 µg/mL) on U251 after 48 h treatment. *Indicates a significant difference at p < 0.05. Data is presented as mean ± S.D, and n = 6. The Y-axis represents cell viability compared to the control. IONPs and DOX-IONPs represent for EDT-IONPs and DOX-EDT-IONPs.

The cytotoxicity of DOX-EDT-IONPs against U251 was studied in comparison to free DOX at different concentrations (0.25, 0.5 and 1 µg/mL). Based on the MTT assay, the cell viability upon a 48 h-treatment with DOX and DOX-EDT-IONPs (at concentration of 1 µg/mL of DOX) decreased to 25% ± 1% and more significantly to 17% ± 2% (p < 0.05), respectively, while no cytotoxic effects of the EDT-IONPs alone observed in the GBM cells (Fig. 5). It should be noted that the IC_50_ of DOX was found to be ca. 300 ng/mL (Fig. 4S), and both concentrations of DOX (either free DOX or DOX-EDT-IONPs) examined (0.5 and 1 µg/mL) were above the IC_50_ value. Based on initial cytotoxic response to the DOX-EDT-IONPs, a 1 µg/mL DOX concentration was selected for the nanoparticle formulations in subsequent studies. Compared to free DOX (1 µg/ml) there was a modest but significant increase in cytotoxicity with the DOX-EDT-IONP. A similar cytotoxic effect of DOX released from DOX-loaded-chitosan-modified Fe_3_O_4_ nanoparticles^12^, and core-shell nanocarriers (ZnO-polyacrylamide-DOX)^44^ against GBM cell lines have previously been reported. In addition to the modest improvement in cytotoxicity of DOX when administered as DOX-EDT-IONPs on GBM cells, the capability of DOX-EDT-IONPs to improve DOX’s delivery across the biological barriers is also of importance in determining overall improvements with the IONP formulation. Moreover, the DOX-EDT-IONPs potentially provide the capability of site-specific magnetic targeting to diminish DOX’s systemic side effects^10,12^. For example, Xu et al.,^10^ reported that IONPs could enhance DOX uptake by C6 glioma cells in rats bearing intracranial tumors particularly in the presence of an external magnetic field, while DOX-associated toxicity was prevented. Correspondingly, animal survival was prolonged from 32 and 38.5 days for mice treated with DOX and DOX-IONPs, respectively to 79.2 days for mice treated with DOX-IONPs in the presence of an external magnetic field.

**Figure 6:**
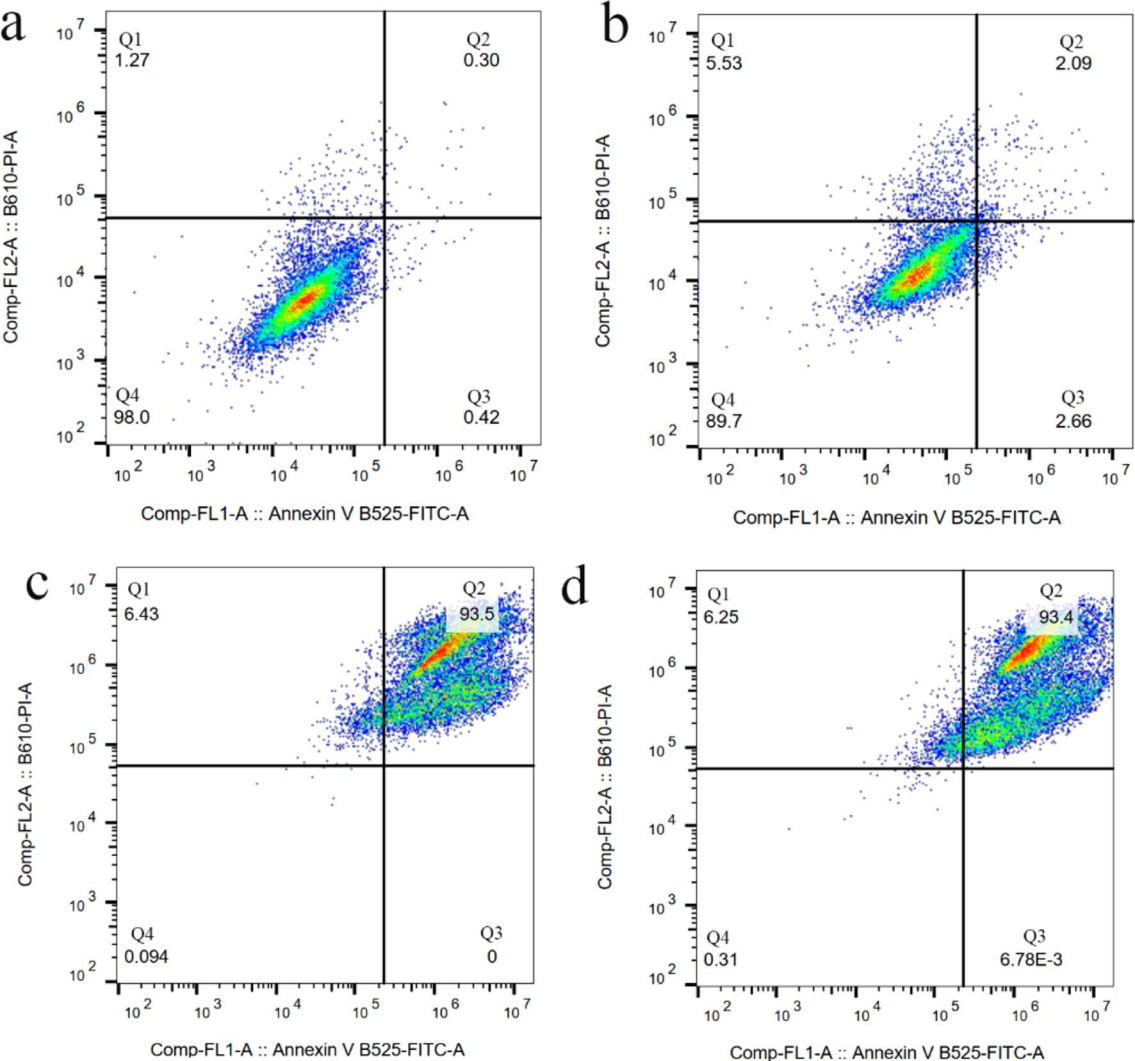
Flow cytometer analysis for cell apoptosis/necrosis of U251 upon 48-h treatment, stained with Annexin V-FITC and PI. (**a**) Control, (**b**) EDT-IONPs, (**c**) DOX, and (**d**) DOX-EDT-IONPs. (Q4) demonstrates Live, (Q3) early apoptotic, (Q2) late apoptotic and (Q1) necrotic cells.

**Figure 7:**
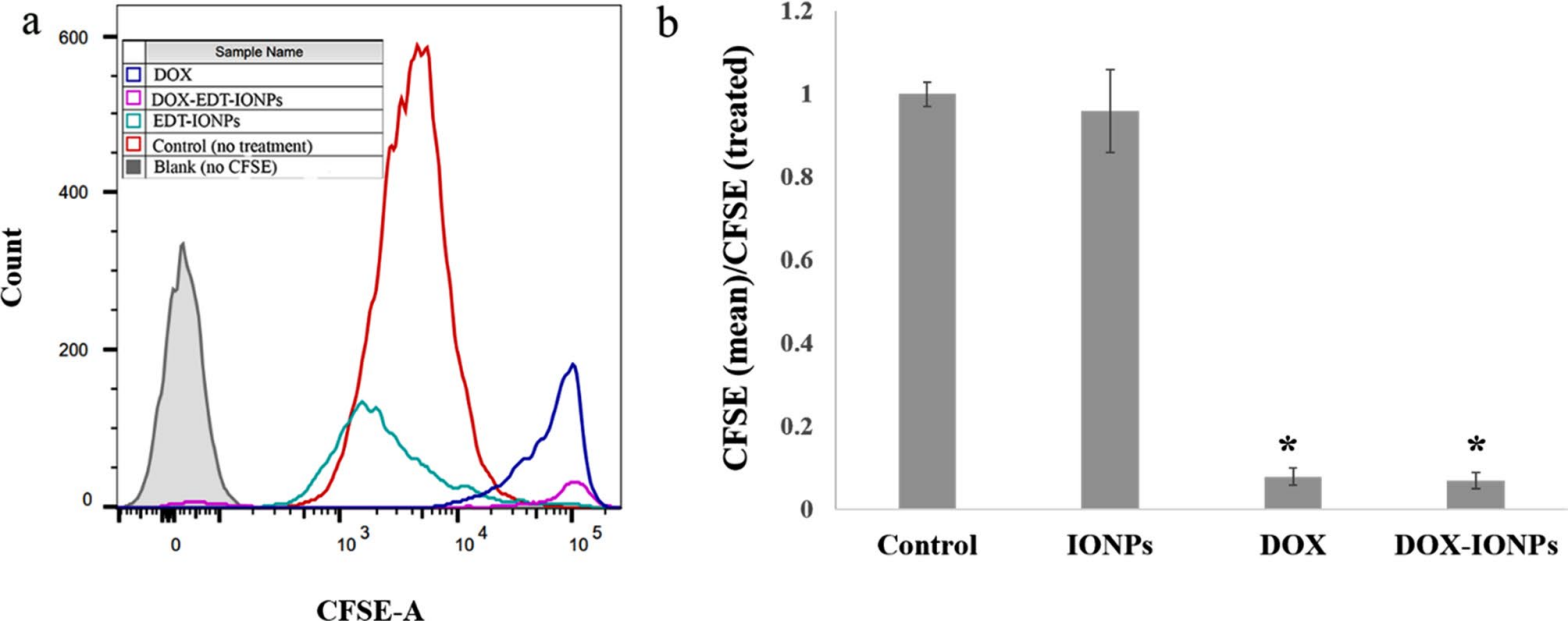
Flow cytometer analysis for cell proliferation assay of carboxyfluorescein succinimidyl ester (CFSE)-labelled U251 upon treatment with EDT-IONPs, DOX and DOX-EDT-IONPs. (**a**) CFSE flow cytometry graph, and (**b**) the relative cell proliferation inhibition calculated by (mean CFSE control/mean CFSE treated). *Shows a significant difference compared to the control group at p < 0.05. IONPs and DOX-IONPs represent for EDT-IONPs and DOX-EDT-IONPs.

Induction of apoptosis through DNA damage via intercalation into DNA and inhibition of topoisomerase-II is a *de facto* mechanism of DOX’s cytotoxicity^45^. In this study, both DOX and DOX-EDT-IONPs were found to be effective at inducing apoptosis in U251 cells after 72 h, leading to ca. 93% late apoptotic cell death, and no considerable cell viability (Fig. 6). In late apoptosis, the cellular membrane integrity is lost, thus cells demonstrate staining with both annexin V-FITC (+)/PI (+) ^46^. Likewise, treatments with both DOX and DOX-EDT-IONPs significantly inhibited GBM cell proliferation by over 90% (Fig. 7). Similarly, the anti-proliferative effects of DOX loaded in catanionic solid lipid nanoparticles (CASLNs)^47^ and DOX-polyglycerol-nanodiamond composites^48^ were reported on U87 GBM cell line. For example, Kuo et al.,^47^ reported a greater anti-proliferative effect of DOX-CASLNs than that of free DOX due to the higher DOX accumulation in GBM cells through a vesicular uptake pathway.

**Figure 8:**
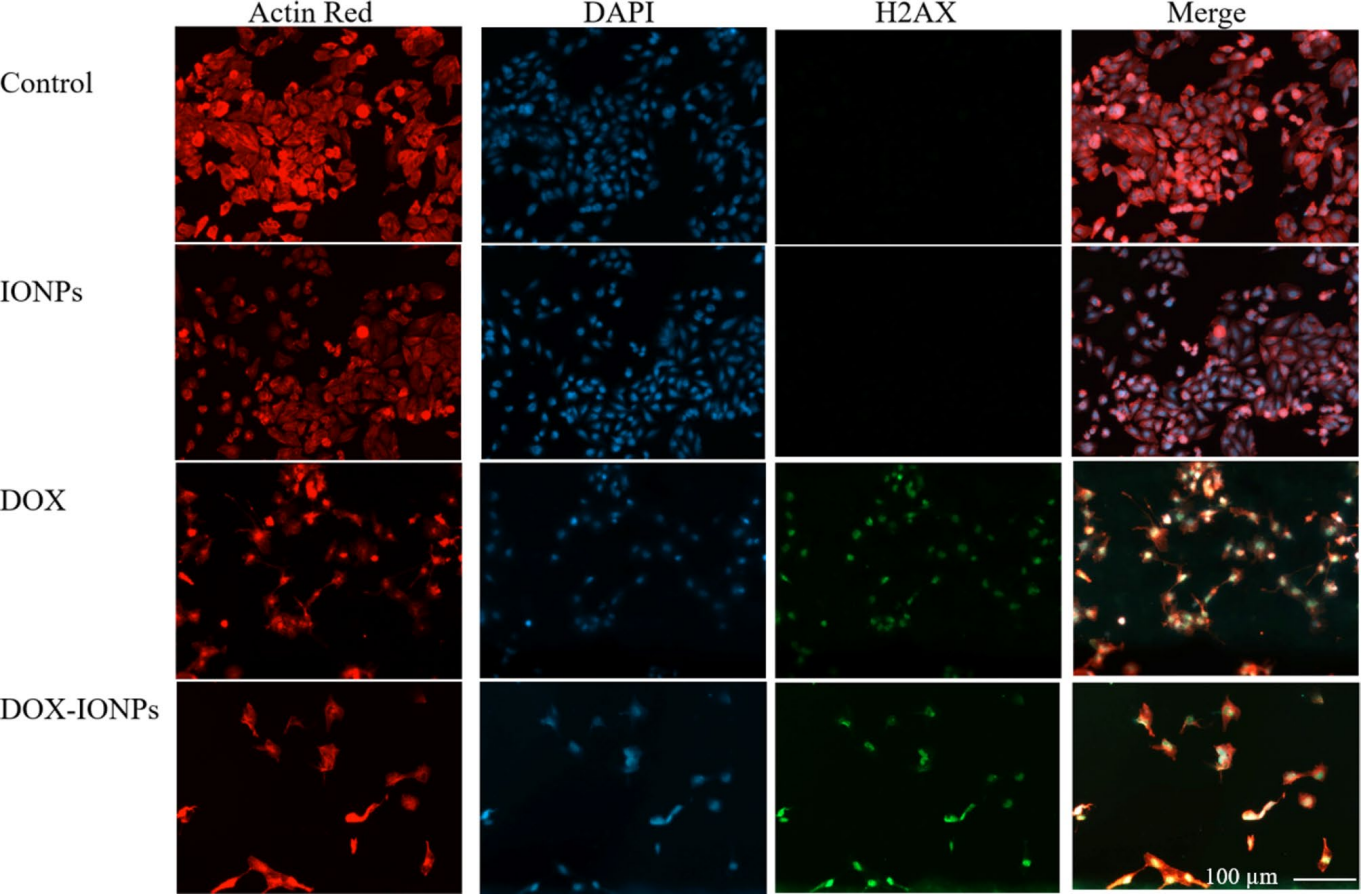
Fluorescence microscopy images of U251 with or without treatment after 48 h. Red, blue and green fluorescence colours represent Alexa Fluor@ 488 phalloidin-stained F-actin, DAPI-stained cell nuclei, and γ-H2AX, a marker of DNA double-strand breaks, respectively. IONPs and DOX-IONPs represent for EDT-IONPs and DOX-EDT-IONPs.

The studies of cell morphology indicated that in addition to a significant reduction in the cell population, both DOX and DOX-EDT-IONPs treatments induced notable morphological changes from a cuboidal morphology of normal U251 to a shrunken and spindle-like structure of actin cytoskeleton and a disrupted nucleus (Fig. 8). The effect of DOX in induction of remodeling in actin cytoskeleton and disruption of central stress fibers leading to impaired cell adhesion and increased cell detachment has been reported previously^49^. Moreover, phosphorylated H2AX (γ-H2AX), mediating DNA double-strand break, is an early and sensitive biomarker in DNA double‐strand break response^50^. In Fig. 8, γ-H2AX can be visualized as foci by immunofluorescence in U251 treated with either DOX or DOX-EDT-IONPs. Such findings indicate DNA damage following DOX treatment in GBM cells. This is in accordance with previous findings of DOX-induced DNA damage and appearance of γ-H2AX in breast^50^ and lung^51^ cancer cells. It is noteworthy to mention that iron oxide nanoparticles typically show quenching effect of DOX fluorescence intensity^52^ and as a result the DOX-EDT-IONPs could not be observed inside the cells by fluorescence microscopy.

The studies of cell morphology indicated that in addition to a significant reduction in the cell population, both DOX and DOX-EDT-IONPs treatments induced notable morphological changes from a cuboidal morphology of normal U251 to a shrunken and spindle-like structure of actin cytoskeleton and a disrupted nucleus (Fig. 8). The effect of DOX in induction of remodeling in actin cytoskeleton and disruption of central stress fibers leading to impaired cell adhesion and increased cell detachment has been reported previously^49^. Moreover, phosphorylated H2AX (γ-H2AX), mediating DNA double-strand break, is an early and sensitive biomarker in DNA double‐strand break response^50^. In Fig. 8, γ-H2AX can be visualized as foci by immunofluorescence in U251 treated with either DOX or DOX-EDT-IONPs. Such findings indicate DNA damage following DOX treatment in GBM cells. This is in accordance with previous findings of DOX-induced DNA damage and appearance of γ-H2AX in breast^50^ and lung^51^ cancer cells.

### ROS generation

**Figure 9:**
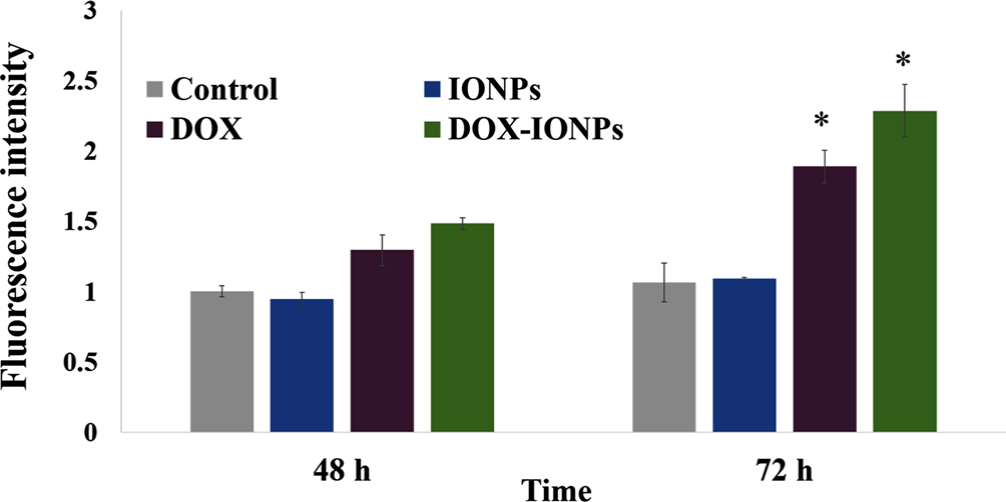
ROS induction by EDT-IONPs, DOX or DOX-EDT-IONPs in U251 at different time-points. *Indicates a significant difference compared to the control group at p < 0.05. Data is presented as mean ± S.D, and n = 5. IONPs and DOX-IONPs represent for EDT-IONPs and DOX-EDT-IONPs.

ROS generation is a well-known mechanism involved in DOX-induced apoptotic death in various cancer cells such as human osteosarcoma Saos-2^53^, and human ovarian cancer cells^54^. In the present study, treatment of U251 with either DOX or DOX-EDT-IONPs increased the formation of ROS by 1.9 ± 0.1 and 2.2 ± 0.2 fold, respectively within 72 h, while only DOX-EDT-IONPs showed a ROS-inducing effect at 48 h (Fig. 9). Similarly, the effect of DOX on ROS induction and apoptosis in U87 GBM cell line has been reported^55^. It is also noteworthy to mention that ROS-induction is one of cytotoxicites associated with bare IONPs and over 800% enhancement in the intracellular ROS was reported into porcine endothelial cells (3 h exposure, 0.5 mg/mL of IONPs)^56^. Nonetheless, our findings indicated that the synthesized EDT-IONPs were biocompatible and did not induce intracellular ROS *per se*. Taken together, triggered ROS-mediated DNA damage is suggested as one of the potential mechanisms of DOX-induced cell apoptosis in human GBM cells.

### Quantitative RT-PCR

**Figure 10:**
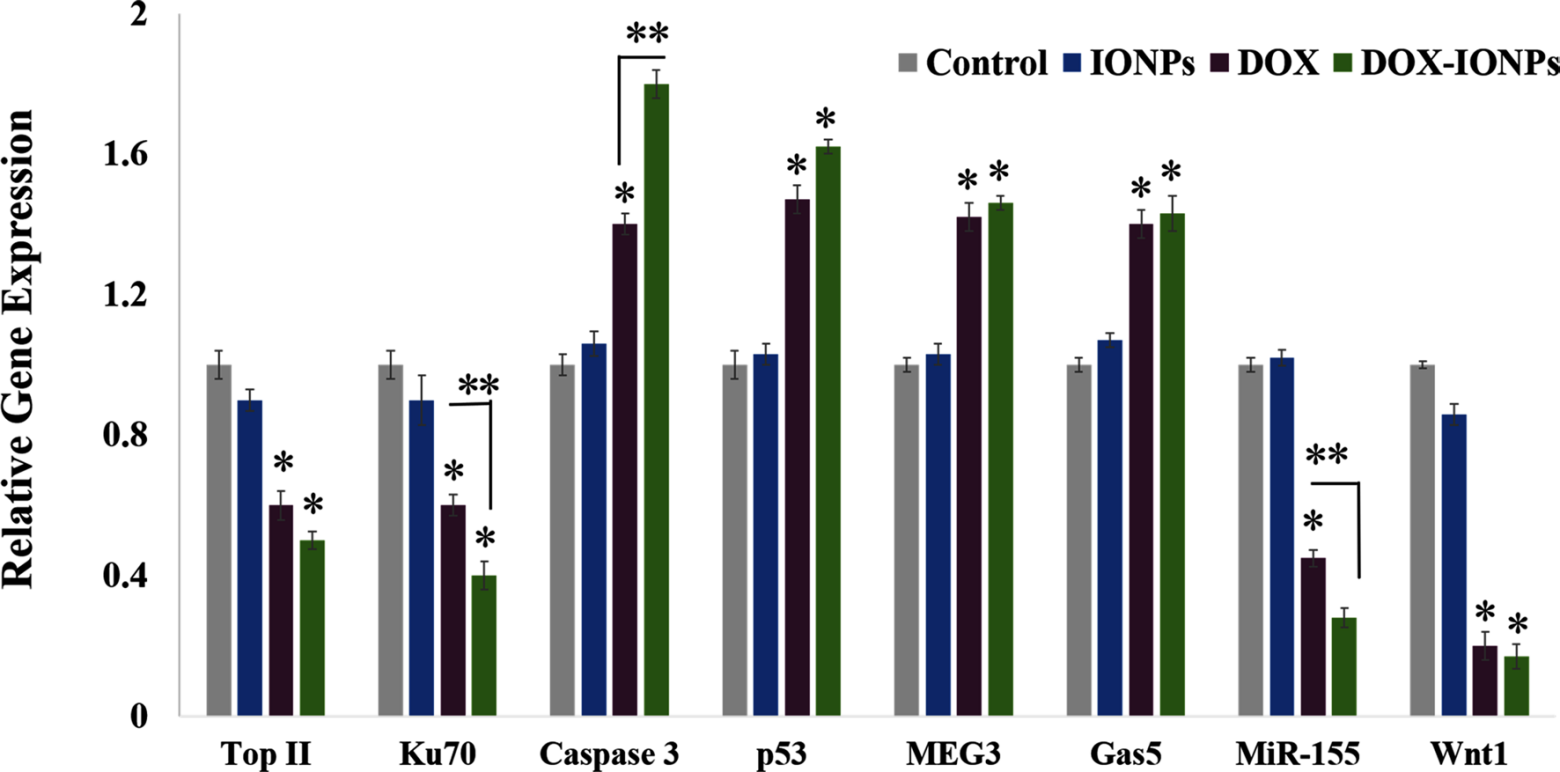
Relative gene expression of U251 cell upon treatment with either EDT-IONPs, DOX or DOX-EDT-IONPs for 48 h. *Indicates a significant difference compared to the control group, and **compared to DOX-treated cells at p < 0.05. Data is presented as mean ± S.D, and n = 5. IONPs and DOX-IONPs represent for EDT-IONPs and DOX-EDT-IONPs.

To demonstrate the anti-cancer effect and mechanism of DOX and DOX-EDT-IONPs treatments on U251, a series of gene studies was conducted (Fig. 10). Topoisomerase IIα (Top II) is a key enzyme in DNA replication, which is considered as a prominent molecular target of several anti-cancer drugs such as DOX and etoposide^57^. DOX inhibits topoisomerase II (TOP2), by which the DNA replication, transcription and repair are interrupted^21^. Ku70, a DNA-dependent protein kinase, is another factor involved in the repair of DNA double-strand breaks and known as a survival factor in some cancer cells^58^. Treatment of U251 with DOX-EDT-IONPs reduced markedly the expression of both Ku70 and Top II, which would further reduce DNA repair and replication in the GBM cells.

Caspases are essential mediators of programmed cell death and they are triggered sequentially, in which activation of Caspase 12 leads to the activation of Caspase 9 and the subsequent ‘effector’ Caspase 3^59^. Both DOX and DOX-EDT-IONPs treatments upregulated the Caspase 3 gene expression, which is consistent with its upregulation in C6 glioma^10^, leukemia HL-60^60^, and MCF-7 breast cancer^61^ cells upon DOX treatments. p53 is a tumor suppressor protein whose mutation is the most prevalent genetic alteration in human cancers^62^. In fact, the p53 protein can inhibit DNA synthesis and regulates cell apoptosis through competition with the DNA repair mechanisms^21^. The U251 cells treated with DOX and DOX-EDT-IONPs exhibited an upregulated expression of p53.

Maternally Expressed Gene 3 (MEG3) is an imprinted non-coding RNA that acts as a tumor suppressor through both p53-dependent and p53-independent pathways^63^. Furthermore, it has been found that MEG3 expression markedly is diminished in glioma tumors, whereas whose expression can inhibit cell proliferation and promoted cell apoptosis in U251 and U87 GBM cell lines^64^. lncRNA-growth arrest-specific 5 (Gas5) is another tumor-suppressor gene that is downregulated in glioma cells^65^. Suppressing the GBM tumor malignancy has been observed through introduction of Gas 5 and consequently downregulation of miR-222^66^. Here, both DOX and DOX-EDT-IONPs treatments were found to be effective in upregulation of both tumor suppressors, i.e. MEG3 and Gas5, which potentially leads to GBM cell apoptosis.

MiR-155 is an important oncogenic microRNA that is overexpressed in various malignant tumors including GBM, whose mechanism of action is associated with a blockade of Caspase-3 activity and regulation of multiple genes involved in cancer cell proliferation, and invasiveness^67^. The expression of MiR-155 in U251 was downregulated upon treatment with either DOX (0.457 ± 0.24 fold) or more significantly with DOX-EDT-IONPs (0.28 ± 0.03-fold, p < 0.05). It also has been reported that downregulation of MiR-155 can enhance the chemosensitivity of U251 cells to Taxol by interrupting the activity of EAG1 pathways and inducing apoptosis^67^.

In addition, the Wnt signaling pathway plays an important role in malignant transformation and tumor progression in gliomas^68^, and the capacity of intracranial tumor formation has been found to be reduced upon Wnt silencing, in vivo^69^. Here, U251 demonstrated a significant downregulation of Wnt1 upon the treatments with either DOX (0.21 ± 0.04 fold) or DOX-EDT-IONPs (0.17 ± 0.03 fold).

### Anti-cancer effect of DOX-EDT-IONPs (in vitro GBM model)

Due to the inability of DOX to cross the BBB and penetrate into the tumor site, it demonstrates little effectiveness in treating GBM when administered systemically^70^. Having considered that, development of an efficient drug delivery system enabling penetration of DOX across the BBB and enhancing its bioavailability is a matter of significant importance in GBM chemotherapy. In selecting the most appropriate cell culture model of the BBB to evaluate the DOX-EDT-IONP delivery approach, both the bEnd.3 brain endothelial cell line and the MDCK-MDR1 cell line were considered. Our previous studies with bEnd.3 indicated this particular model was well suited for examining nanoparticle permeability^71^. However, the available brain endothelial cell culture models do not form a restrictive paracellular barrier required for screening the passage of small molecules^72^ and because of this often overestimate the BBB penetration. The MDCK-MDR1 cells overexpress P-gp and have reduced paracellular diffusion of solutes due to the complex tight junction proteins. For this reason, the MDCK-MDR1 cell line is often used to assess the BBB permeability and P-gp liabilities of drugs for central nervous system indications^73^. Since the penetration of DOX across the BBB is mainly restricted by the P-gp expression under normal physiological conditions^74^, the MDCK-MDR1 cells overexpressing P-gp were used in the present study to provide a more representative barrier cell for the BBB-GBM co-culture model.

**Figure 11:**
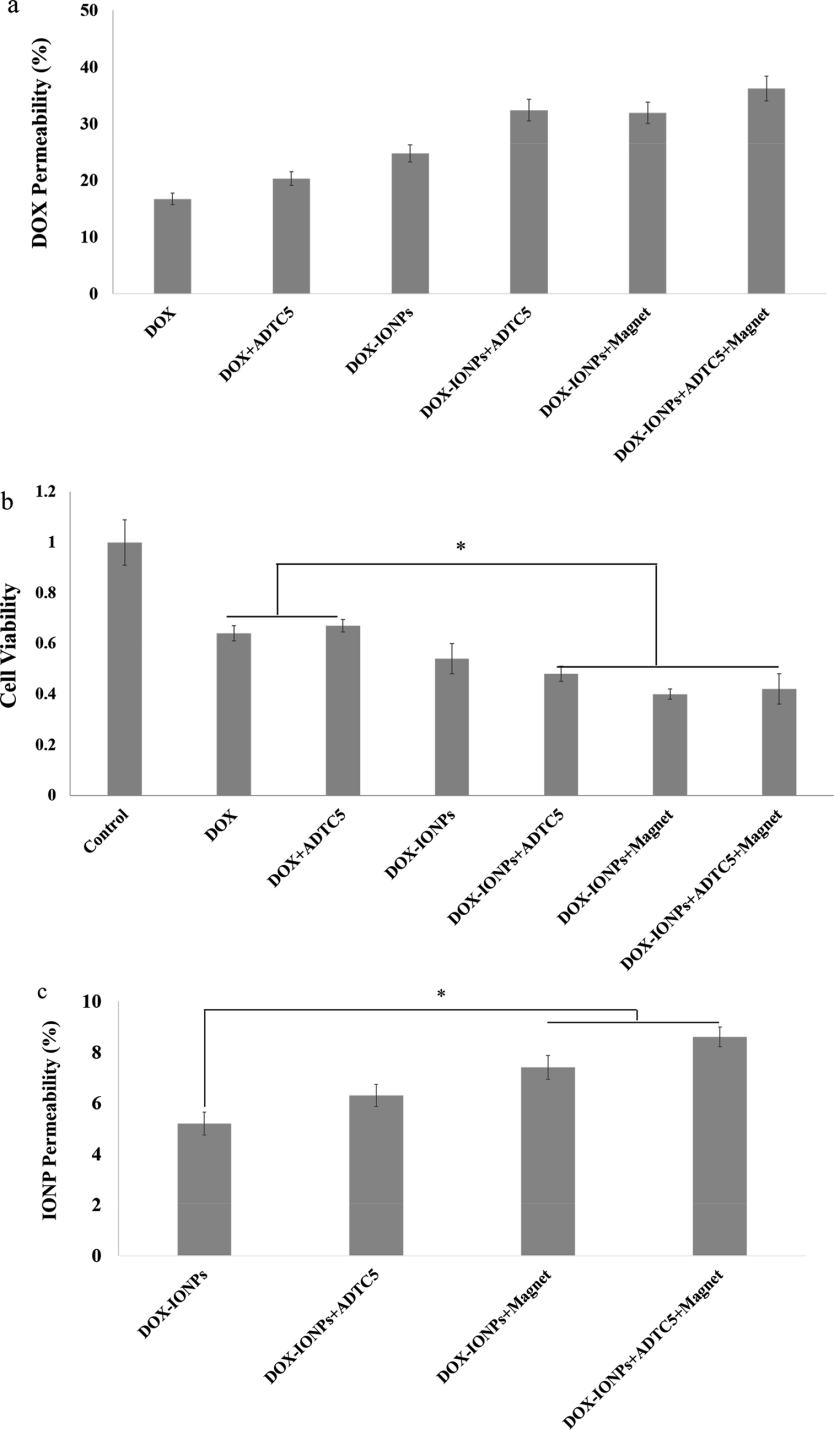
Anti-cancer efficacy of DOX-EDT-IONPs compared to the free DOX on an MDCK-MDR-GBM model in vitro. (**a**) DOX permeability across the MDCK-MDR1 monolayer with or without IONPs in the presence or absence of magnet and ADTC5 (**b**) cytotoxicity of each formulation on U251 cells after penetrating the monolayer. (**c**) DOX-IONPs permeability across the MDCK-MDR1 monolayer with or without magnet and ADTC5. *Indicates a significant difference at p < 0.05 with the other treated groups. Data is presented as mean ± S.D, and n = 3. IONPs and DOX-IONPs represent for EDT-IONPs and DOX-EDT-IONPs.

The limited penetration of DOX was apparent in the BBB-GBM co-culture model (Fig. 11a). The DOX permeability across the MDCK-MDR1 monolayers reached approximately 18% after 4 hours. The DOX that penetrated the MDCK-MDR1 monolayers had reduced cytotoxicity compared to that observed with 1 μg/ml of DOX in U251 monocultures. The DOX-EDT-IONP formulation showed a significant increase (ca. 1.5-fold) in penetration of DOX compared to DOX alone in the BBB-GBM co-culture model (Fig. 11a). As DOX is a substrate of P-gp efflux transporter that highly restricts its penetration into the brain^74^, the improvement in DOX permeability observed in the present study is attributed to the DOX-loaded nanoparticles circumventing the P-gp efflux transporter in the MDCK-MDR1 monolayers. However, despite the increase in permeability observed with DOX-EDT-IONP in the in vitro BBB-GBM co-culture model, the resulting cytotoxicity in U251 was not significantly greater than that of DOX alone (Fig. 11b). This suggests that improvement in DOX permeability with the IONP formulation alone was not sufficient to produce an enhanced cytotoxic response and additional measures were necessary to impact both permeability and response in the BBB-GBM co-culture model.

Transient disruption of the BBB with hyperosmotic solutions like mannitol has been reported to enhance the delivery of therapeutic molecules as well as IONPs into the brain^75,76^. In this regard, mannitol has extensively been used in combination with anti-tumor agents in clinical trials of glioma therapy over the last three decades^77^. Similarly, Sun et al.,^78^ reported a significant increase in permeability of both EDT-IONPs and aminosilane-coated (AmS)-IONPs across brain endothelial cell monolayers when tight junctions were disrupted using mannitol. However, the extensive opening of the BBB by mannitol and the long recovery time for re-establishment of the BBB integrity can cause a substantial and uncontrolled influx of low and high molecular weight substances from the blood into the brain that can result in neurological toxicity, jeopardizing patient safety^79^. Previous studies using cadherin binding peptides to modulate BBB permeability suggested a more controlled opening of the BBB in terms of both magnitude and duration of opening was possible^80^. In the present study, the cadherin binding peptide, ADTC5 was used to transiently modulate permeability in the BBB-GBM co-culture model. While ADTC5 was able to increase the permeability of the DOX-ECT-IONPs, especially when combined with an external magnetic field (Fig. 11), wholesale disruption of monolayer integrity was much less than observed with hyperosmotic mannitol, as demonstrated by the permeability to the 35 kDa molecular weight IRDye [2.7 ± 0.4% and 3.1 ± 0.3% without and with ADTC5, respectively, compared to 15.6 ± 0.6% with mannitol (Fig. 5S)]. Moreover, as mentioned previously, the recovery time for re-establishment of the BBB integrity was reported to be within 60 min post-injection of the cadherin binding peptide in vivo^36^. This means that using the cadherin peptide allowed the MDCK-MDR1 monolayers to maintain barrier properties to large IRDye macromolecule marker, while allowing enhanced penetration to the IONPs, especially in the presence of an external magnetic field. We have named this approach magnetic enhanced convective diffusion (MECD) as the IONPs diffuse across the transiently disrupted cell barrier in a bulk flow manner that is accelerated by the presence of an external magnetic field.

By transiently opening the MDCK-MDR1 monolayer tight junctions using ADTC5 and in combination with an external magnetic field, the GBM cell viability significantly decreased upon treatment with DOX-EDT-IONPs compared to the GBM cells treated with free DOX (cell viability 66 ± 3.3% and 45 ± 3.7% for GBM cells treated with free DOX and DOX-EDT-IONPs, respectively) (Fig. 11b). This result was consistent with the higher DOX-EDT-IONP permeability through the MDCK-MDR1 monolayer when both ADTC5 and external magnetic fields were applied.

The permeability of DOX-EDT-IONPs through BBB-GBM co-culture model was also examined. Under normal conditions, DOX-EDT-IONPs showed 5.2 ± 0.4% penetration across the MDCK-MDR1 monolayer over 4 hours. The diffusion of DOX-EDT-IONPs could be increased by either enhancing the MDCK-MDR1 monolayer permeability with ADTC5 (6.2 ± 0.45%) or by application of an external magnetic field (7.4 ± 0.5%) (Fig. 11c). However, combining both ADTC5 treatment with an external magnetic field significantly augment DOX-EDT-IONP penetration by 8.5 ± 0.36%. To the best of our knowledge, this is the first report on the combinational effect of cadherin binding peptide and external magnetic field as an effective approach to enhance the permeability of drug delivery systems across the BBB.

As mentioned earlier, IONPs uniquely provide a site-specific magnetic targeting utilizing an external magnetic field to draw the nanoparticles to the site of action and enhancing their bioavailability^81^. For instance, by applying an external magnetic field, overall tumor exposure to magnetic nanoparticles was enhanced by 5-fold compared to non-targeted tumors^82^. Moreover, ADTC5 has shown an enhanced delivery of various marker molecules (e.g., ^14^C -mannitol, Gd-DTPA) across the MDCK monolayer *in vitro*, and the BBB *in vivo* through binding to the EC1 domain of E-cadherin, blocking the cadherin–cadherin interactions and thus enhancing the delivery of molecules into the brain via the paracellular pathway of the BBB^80^.

Therefore, the developed DOX-EDT-IONPs in combination with the magnetic enhanced convective diffusion and the cadherin binding peptide for transiently opening the BBB tight junctions were found effective to enhance DOX’s bioavailability and anti-cancer effect in GBM cells by virtue of overcoming the MDR and enhancing the permeability of DOX through a BBB model in vitro*.*

This combinational approach can potentially be an efficacious alternative for the passive targeting through the enhanced permeability and retention (EPR) effect, or ligand-based active targeting of the IONPs in clinical practice. In fact, the EPR effect in humans has been found not as prominent as in animal models^83^. Moreover, during early stages of brain tumor development, the EPR effect cannot play an important role inasmuch as the BBB is still intact, and leakiness is observed at the stages when tumor volume is high and difficult to treat^84^. In addition, the infiltrating tumor cells are mostly associated with the intact BBB that would impede passive targeting of nanoparticles^85^. On the other hand, the clinical outcomes of active targeting of nanoparticles to brain tumors have not yielded the results anticipated due to altered expression of target receptors in some types of tumors, tumor heterogeneity and the interpatient variability^86^. BIND-014 and MM-302 are two examples of active targeting nanomedicines that failed in clinical studies^87^. In light of these clinical studies, this novel combinational approach of using cadherin binding peptide for transiently opening the BBB tight junctions in juxtaposition with magnetic enhanced convective diffusion can be an alternative and effective approach for the passive targeting and ligand-based active targeting of drug-loaded IONPs in clinical practice. This combinational approach can provide a site-specific magnetic targeting to reduce systemic distribution of the drug-loaded IONPs, a transiently opening of the BBB tight junctions using a cadherin binding peptide, and an enhanced convective diffusion of the magnetic nanoparticles into the brain. These together can reduce the systemic toxicity of chemotherapy, enhance the permeability of the drug-loaded nanoparticles into the brain and ameliorate the efficacy of GBM chemotherapy by providing a therapeutic concentration of the effective anti-cancer drugs like DOX that are intrinsically impermeable to the BBB.

## Conclusion

In this study, DOX-EDT-IONPs were developed to facilitate drug delivery to GBM tumor cells. The DOX was entirely released from the DOX-EDT-IONPs within 4 days, while the nanoparticles could augment the DOX’s uptake in U251 cells by 2.8-fold. The DOX-EDT-IONPs was found to be effective in apoptosis-induced cell death, proliferation inhibition, and ROS-induction in U251 cells. Moreover, DOX-EDT-IONPs treatment could downregulate TOP II and Ku70, essential enzymes for DNA repair and replication, as well as MiR-155 oncogene, while concomitantly upregulated Caspase 3, a key mediator of apoptosis, and tumor suppressors i.e., p53, MEG3 and GAS5, in U251 cells. Furthermore, recruiting an in vitro MDCK-MDR-GBM co-culture model, the EDT-IONPs could enhance DOX penetration through the MDCK-MDR1 monolayer over twofold and provided significantly higher anti-cancer effect than free DOX in GBM cells in the presence of an external magnetic field and ADTC5. In addition, the DOX-EDT-IONPs showed increased permeability through the MDCK-MDR1 monolayer that was further significantly increased in the presence of an external magnetic field. The results of these studies suggest magnetically driven enhanced diffusion of DOX-EDT-IONPs across the MDCK-MDR1 monolayer. However, the largest increase in DOX-EDT-IONP permeability in the BBB-GBM co-culture model was observed using a cyclic ADT peptide as a transient disruption agent in combination with an external magnetic field. The combination of an external magnetic field and a cadherin binding peptide augmented the penetration of the DOX-EDT-IONPs, under conditions that did not extensively disrupt the BBB as compared to other techniques like hyperosmotic treatment. If similar effects are observed in vivo, the advantage of this approach would be that the BBB remains relatively intact to large macromolecules, which correspondingly should diminish the risk of neurological toxicity. In conclusion, the developed DOX-EDT-IONPs in combination with the magnetic enhanced convective diffusion and the ADTC5 for transiently opening the BBB tight junctions can potentially provide an efficacious formulation of DOX in GBM chemotherapy by virtue of enhancing DOX’s penetration into the brain, overcoming the MDR cancer cells, providing a site-specific magnetic targeting and diminishing the systemic toxicity.

## Materials and methods

### Materials

The chemical reagents were acquired from Sigma Aldrich (St. Louis, MO), and the cell culture and biochemical reagents were purchased from Thermo Fisher Scientific Inc, USA, unless otherwise specified.

### Synthesis and characterization of EDT-IONPs

Iron oxide nanoparticles were fabricated under mild conditions at room temperature as previously described^31^. Briefly, Fe(acac)_3_ (2.83 g, 8 mmol) was dissolved in ethanol/DI water (6:4) and purged with nitrogen for 1 h, followed by adding NaBH_4_ (3.03 g, 80.0 mmol) in deoxygenated DI water under stirring (1000 rpm). When the color of the reaction mixture changed from red to black, it indicates the formation of IONPs (approximately 20 min). For coating, (Trimethoxysilylpropyl)-ethylenediamine triacetic acid (EDT, 16 ml) was added, and the reaction mixture was stirred overnight at room temperature. The blackish brown solution was filtered, and the solvent was evaporated at 50 °C under low pressure. The obtained viscous mixture was dissolved in 200 ml of cold ethanol and left until excess NaBH_4_ became crystallized, which was removed by filtration. Finally, ethanol was completely removed, the product was dissolved in 50 ml DI water and dialyzed against DI water to remove the unreacted EDT, followed by centrifugation at 4000 rpm for 30 min^31^. The dark reddish-brown supernatant was collected and stored for further use.

The size distribution of EDT-IONP in DI water was measured by dynamic light scattering (DLS) using a Photocor Complex system. The FTIR spectrum was taken using a Thermo Nicolet iS10 FTIR spectrometer. Transmission electron microscopy (TEM) images of the EDT-IONPs were obtained using a Philips CM 10 electron microscope (FEI, Hillsboro, USA).

### Drug loading on EDT-IONPs

To load DOX on the EDT-IONPs, EDT-IONPs (20 µg) and DOX (20 µg) in 200 µL phosphate-buffered saline (PBS, pH 6) was combined and incubated overnight under ambient conditions. Afterwards, the mixture was centrifuged at 12,000 rpm for 10 min and the solution was completely withdrawn. Then, the nanoparticles were washed with PBS (pH 7.4) twice to remove free DOX and the nanoparticles were centrifuged again to collect the DOX-loaded EDT-IONPs (DOX-EDT-IONPs).

### Biocompatibility assessment of EDT-IONPs

To assess the biocompatibility of the synthesized EDT-IONPs, a mouse brain-derived microvessel endothelial cell line, bEnd.3 (American type tissue culture collection, Manassas, VA) was employed as a cell culture model for the BBB. The Madin–Darby canine kidney (MDCK) transfected with multi-drug resistant protein 1 (MDR) was also used. MDCK is an epithelial cell line originally derived from the normal dog kidney and transfected with MDR, expressing P-gp and tight junction proteins. Therefore, the MDCK-MDR1 has been reported as a model for the BBB permeability^73^. Furthermore, an authenticated human U251 GBM cell line was used for biocompatibility evaluation of EDT-IONPs. The bEnd.3, MDCK-MDR1 and U251 cells (cells at passage number 20–30) were cultured at a density of 2 × 10^4^ (bEnd.3, MDCK-MDR) and 1 × 10^4^ (U251) cell/cm^2^ in 96-well plates, and incubated overnight at 37 °C allowing the cells to attach. Next day, the cells were treated with EDT-IONPs (0.25 to 50 µg/mL) suspended in the cell culture medium for 48 h. Thereafter, the culture medium was removed, and the cells were washed with PBS followed by incubation with fresh medium containing 0.5 mg/mL of 3-(4,5-dimethylthiazol-2-yl)-2,5-diphenyltetrazoliumbromide (MTT, 0.5 mg/mL) reagent at 37 °C. After 3 h, the medium was withdrawn, and blue crystals were dissolved in pure DMSO^88–90^. The absorbance of the solutions was measured using a Synergy HT plate reader (BioTek, Winooski, VT) at the wavelength of 570 nm and the relative cell viability was calculated as [OD]_test_/[OD]_control_, upon five measurements.

### Drug release from EDT-IONPs

The release of DOX from the EDT-IONPs was measured in PBS (pH 7.4 mimicking physiological pH, and 4.5 mimicking pH of acidic intracellular compartments such as endosomes) at 37 °C. For this purpose, the DOX-EDT-IONPs were suspended in 1 mL PBS in Eppendorf tubes and at various time points, the tubes were centrifuged at 12,000 rpm for 10 min to pellet the nanoparticles and the solution was completely collected followed by re-suspension of the nanoparticles in 1 mL of fresh PBS. The concentration of the released DOX in the solution was determined by fluorescence measurement (excitation and emission wavelengths of 485 nm and 590 nm, respectively) using a Synergy HT plate reader. The concentration of the released DOX from DOX-EDT-IONPs was calculated using a serial dilution of a DOX standard solution.

### Cellular uptake of EDT-IONPs and DOX

To study the cellular uptake of DOX-EDT-IONPs; bEnd.3, MDCK-MDR, and U251 cells were grown in 24-well culture plates to reach a confluent monolayer and then they were treated with cell culture medium containing either EDT-IONPs or DOX-EDT-IONPs (10 and 20 µg/mL) for 4 h at 37 °C with and without a static magnetic field (rare-earth circular magnets, diameter: 20 mm, Lee Valley, Winnipeg, CA). Then the cells were washed with cold PBS to remove non-adhered nanoparticles, and lysed with 0.1% triton solution in PBS overnight at − 20 °C. The content of IONPs was determined based upon the Ferrozine assay as previously reported^38^. Briefly, HCl (500 µL of 12 M) was added to wells, and were incubated at room temperature for 1 h with gentle shaking to digest the IONPs, followed by neutralization with NaOH (500 µL of 12 M). Then, hydroxylamine hydrochloride (120 µL of 2.8 M) in 4 M HCl was added, and the samples were incubated for 1 h at room temperature with gentle shaking. Afterwards, ammonium acetate solution (50 µL of 10 M, pH 9.5) and ferrozine (300 µL of 10 mM) in 0.1 M ammonium acetate solution were added sequentially to each well, and the absorbance of the solutions was determined at 562 nm by a Synergy HT plate reader. The concentration of EDT-IONPs was quantified based upon an iron chloride standard solution. The protein content of the lysed cells was also measured using a BCA protein assay kit.

The localization of EDT-IONPs in the cell organelles was also studied using TEM as previously described^29,42^. For this purpose, U251 cells were treated with either EDT-IONPs or DOX-EDT-IONPs in accordance with the uptake study, and after washing with PBS, the cells were disassociated using a 0.25% trypsin EDTA solution (Hyclone, Logan, UT). After centrifugation of the collected cells (5 mins at 1500 g), the cell pellet was resuspended in 3% glutaraldehyde in 0.1 M phosphate buffer (pH 7.3) for 3 hours at room temperature. Then the samples were fixed for 2 h at room temperature in 1% osmium tetroxide in 0.1 M phosphate buffer, dehydrated in ascending concentrations of ethanol and embedded in Epon resin. Thin sections were stained with uranyl acetate and lead citrate, and photographed by TEM.

To measure the cellular uptake of DOX, U251 cells were grown in 6-well plates as described earlier and treated with cell culture media supplemented with an equal drug concentration of either DOX or DOX-EDT-IONPs to initiate the cellular drug accumulation. After 2 h, the cells were washed with cold PBS three times and lysed with 0.1% triton solution in PBS as described somewhere else^91^. The concentration of DOX in the cell lysates was measured as delineated in section “Drug release from EDT-IONPs” and normalized with the protein content of the lysed cells.

### Cytotoxicity of DOX-EDT-IONPs in GB cell line

The cytotoxicity of DOX-EDT-IONPs against U251 cells was studied using MTT and flow cytometry analyses. For MTT assay, the cells were cultured as described in section “Cytotoxicity of DOX-EDT-IONPs on cancer cell”. Next day, the medium was changed with fresh medium (negative control), medium containing free DOX with equivalent concentrations corresponding to DOX released from EDT-IONPs at the same period of time (positive control), EDT-IONPs and DOX-EDT-IONPs. After a 48-h treatment, viability of the cells was determined by MTT assay as described in section “Biocompatibility assessment of EDT-IONPs”.

Moreover, cell apoptosis/necrosis was investigated using Annexin V-FITC/PI apoptosis Kit. For this study, the cells were treated with either EDT-IONPs, free DOX or DOX-EDT-IONPs over a 48-h period, followed by incubation in fresh cell culture media without any treatment for 24 h. Afterwards, the cells were stained with Annexin V-FITC and PI in accordance with the manufacturer’s protocol, and consequently were analyzed using flow cytometry (BD FACSCanto II Flow Cytometer instrument (BD Bioscience)). In addition, to study the effect of various treatments on cell proliferation, U251 cells were stained with a fluorescent carboxyfluorescein succinimidyl ester dye (CFSE, 50 mM), for 20 min at 37 °C. Thereafter, the medium was removed, and the cells were washed and treated with either free DOX, EDT-IONPs, or DOX-EDT-IONPs for 48 h followed by changing the media and leaving the cells without further treatment for 24 h. Then, the fluorescence intensity of the cells was determined using flow cytometry. In fact, during each cell division, the cellular content of CFSE decreases that results in a sequential halving of the cellular fluorescent intensity with each mitotic event^92^.

To observe any changes in morphology, the U251 cells were treated for 48 h as mentioned above, followed by washing with PBS, fixating with paraformaldehyde (4% v/v) for 20 min at room temperature and permeabilization with Triton X-100 (0.2% v/v) for 10 min. The specimens were then blocked with BSA solution (3% w/v) for 1 h at room temperature, washed with PBS, and the cells incubated with primary phosho-H2AX antibody solution (1:500 in 3% BSA, 0.3% Triton X-100 in PBS) at 4°C overnight. Afterwards, the primary antibody was withdrawn, and a goat anti-rabbit secondary antibody labeled with Alexa 488 dye (1:500 in the same buffer as the primary antibody) was added to each well and incubated at room temperature for 1 h. Then, the cells were washed with PBS and the actin cytoskeleton was stained with ActinRed for 30 min followed by the nucleus staining with DAPI solution (100 nM) for 5 min at 37 °C. Finally, the samples were washed with PBS and visualized by a fluorescence microscope (Zeiss Axio observer Z1, Germany).

### Reactive oxygen species measurement

The extent to which the various treatments resulted in ROS generation in the U251 cells was evaluated via the peroxide-dependent oxidation of the non-fluorescent 2′,7′-dichlorofuorescein diacetate (DCFDA). In this cell-based assay, DCFDA freely diffuses into the cells. Once inside, the DCFDA is transformed to the highly fluorescent and cell impermeable 2′,7′-dichlorofluorescein (DCF) through ROS mediated metabolism^93^. For this study, the cells were cultured in black 96 well plates at a density of 5000 cell/cm^2^. Next day, the cells were washed with PBS and exposed to 50 μM DCFHDA in PBS for 45 min at 37 °C. Afterwards, the DCFHDA solution was removed, and the cells were washed and treated with either EDT-IONPs, DOX or DOX-EDT-IONPs in cell culture media over 72 h. At various time points, cellular accumulation of ROS in response to the treatments was calculated by measuring the oxidation of DCFDA to the fluorescent DCF using a Synergy HT fluorescent plate reader at Ex/Em 485/590 nm.

### Quantitative RT-PCR

The gene studies were conducted on U251 cells upon a 48-h treatment with either EDT-IONPs, DOX or DOX-EDT-IONPs. To this end, total RNA of the cells was extracted using TRIZOL reagent (Invitrogen, USA) according to the manufacturer’s protocol. Then, the purity and concentration of the extracted RNA were determined by UV-VIS spectrophotometry (NanoDrop, Thermo Fisher Scientific Inc, USA). Afterwards, the level of mRNA encoding Top II, Ku70, p53, Caspase 3, Wnt 1, MEG3, GAS5, and MIR155 was determined by quantitative reverse-transcript polymerase chain reaction (qRT-PCR). The RT-PCR was implemented using iTaq Universal SYBR Green Supermix kit (Bio-Rad, USA) in an Applied Biosystems 7300 Real-Time PCR system and β-actin was employed as the housekeeping gene. The following thermal cycles were designed for the reactions: 1 cycle of 10 min at 50 °C for the reverse transcription reaction, 1 cycle of 1 min at 95 °C for polymerase activation, 40 cycles consisting of 15 s at 95 °C for denaturation and 1 min at 60 °C for annealing. The expression of the target genes was normalized to the β-actin expression and relative gene fold changes were calculated using the comparative C_t_ method (2^−ΔΔCt^) as mentioned previously^88^. The primer sequences are shown in Table 1.

**Table 1:** Sequences of human primers.

	Forward	Reverse
TOP2	ATTCCCAAACTCGATGATGC	CCCCATATTTGTCTCTCCCA
Ku70	CTGTCCAAGTTGGTCGCTTC	CTGCCCCTTAAACTGGTCAA
p53	TCTGAGTCAGGCCCTTCTGT	GTTCCGAGAGCTGAATGAGG
Caspase 3	CTCTGGTTTTCGGTGGGTGT	CGCTTCCATGTATGATCTTTGGTT
Wnt1	CAACAGCAGTGGCCGATGGTGG	CGGCCTGCCTCGTTGTTGTGAAG
GAS5	TGGTTCTGCTCCTGGTAACG	AGGATAACAGGTCTGCCTGC
MEG3	GCTGAAGAACTGCGGATGGA	CATTCGAGGTCCCTTCCCAC
MIR 155	AATCGTGATAGGGGTTTTTGCC	ATGTAGGAGTCAGTTGGAGGC
β-actin	AATGCCAGGGTACATGGTGG	AGGAAGGAAGGCTGGAAGAGTG

### In vitro BBB-GBM model

Nanoparticles as a drug carrier for brain tumor therapy need to first overcome the limited permeability of the BBB as well as the efflux transporters such as P-gp expressed on the brain endothelial cells, which are responsible for low drug permeation into the brain. The Madin–Darby canine kidney epithelial cell line stably transfected with human multi-drug resistant protein 1 (MDCK-MDR) cells overexpress P-gp, and have reduced paracellular diffusion due to the complex tight junction proteins. Together these properties make MDCK-MDR1 cells a reproducible and accurate in vitro cell culture model for examining and predicting the penetration of drugs and solutes across the BBB^73^. In the present study, MDCK-MDR1 cells (passage number 20–30, cell density 100,000 cell/cm^2^ were plated on the apical side of a porous polycarbonate membrane inserts (4.6 cm^2^ pore size: 3.0 μm, Corning Inc., USA). Once a confluent MDCK-MDR1 monolayer was obtained (typically in 6 days), U251 cells were cultured in the basolateral side of the well plates. Free DOX (1 µg/mL) or DOX-EDT-IONPs was added to the apical media compartment of the insert along with an IRdye 800CW PEG as a permeability marker. In addition, a cyclic ADTC5 peptide (Cyclo(1,7)Ac-CDTPPVC-NH_2_), which was synthesized as previously reported^94^ was added to the apical media compartment of the insert to block the cadherin–cadherin interactions and thus enhancing drug delivery through the MDCK-MDR1 monolayer. The cells were then incubated at 37 °C for 4 h in both the presence and absence of a static magnetic field (rare-earth circular magnet, diameter: 30 mm, Lee Valley, Winnipeg, CA). Afterwards, the apical media and the inserts were removed and the GBM cells with the basolateral cell culture media were incubated for an additional 48-h after which the basolateral media was collected to determine IONP (Ferrozine assay) and IR dye permeability as well as the cell viability (MTT assay).

### Statistical analysis

The studies were conducted in triplicate and the results were reported as the mean ± standard deviation (SD). Statistical analysis was conducted using analysis of variance (ANOVA) and p < 0.05 was considered as the criterion of significance, as previously reported.

## Supplementary information

Supplementary information

## Data Availability

The datasets produced during and/or analysed during the current study can be available from the corresponding author on reasonable requests.
