# Supplementary material for: Doxorubicin-loaded iron oxide nanoparticles for glioblastoma therapy: a combinational approach for enhanced delivery of nanoparticles
Source: Sci Rep. 2020 Jul 9;10:11292. doi: 10.1038/s41598-020-68017-y (PMC7347880; doi:10.1038/s41598-020-68017-y)
Supplement: Supplementary file 1 — Supplementary information [file 41598_2020_68017_MOESM1_ESM.docx]

# **Doxorubicin-loaded Iron Oxide Nanoparticles for Glioblastoma Therapy: A Combinational Approach for Enhanced Delivery of Nanoparticles**

Mohammad Norouzi^1,2^, Vinith Yathindranath^2^, James A. Thliveris^3^, Brian M. Kopec^4^, Teruna J. Siahaan^4^, Donald. W Miller*^1, 2^

1. Department of Biomedical Engineering, University of Manitoba, Winnipeg, MB, Canada.
2. Department of Pharmacology and Therapeutics, University of Manitoba, Winnipeg, MB, Canada.
3. Department of Human Anatomy and Cell Science University of Manitoba, Winnipeg, MB, Canada.
4. Department of Pharmaceutical Chemistry, University of Kansas, Lawrence, KS, USA

Corresponding author: Miller, D.W. ([donald.miller@umanitoba.ca](mailto:donald.miller@umanitoba.ca))

Department of Pharmacology & Therapeutics Kleysen Institute for Advanced Medicine

A205 Chown Bldg., 753 McDermot Avenue, University of Manitoba

Tel: +1-204-789-3278

Supplementary


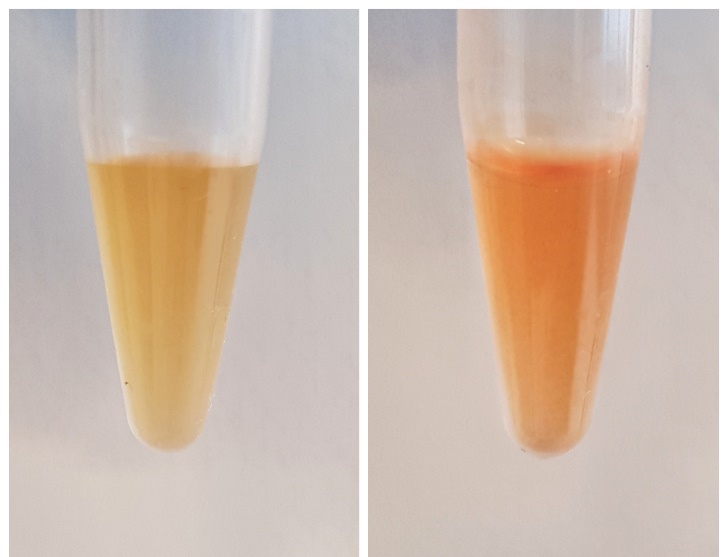


Fig. 1S. Photographs of EDT-IONP (left) and DOX- EDT-IONP (right) solutions in PBS. Both suspensions were stable and the reddish of the DOX-EDT-IONP is attributed to the presence of DOX.


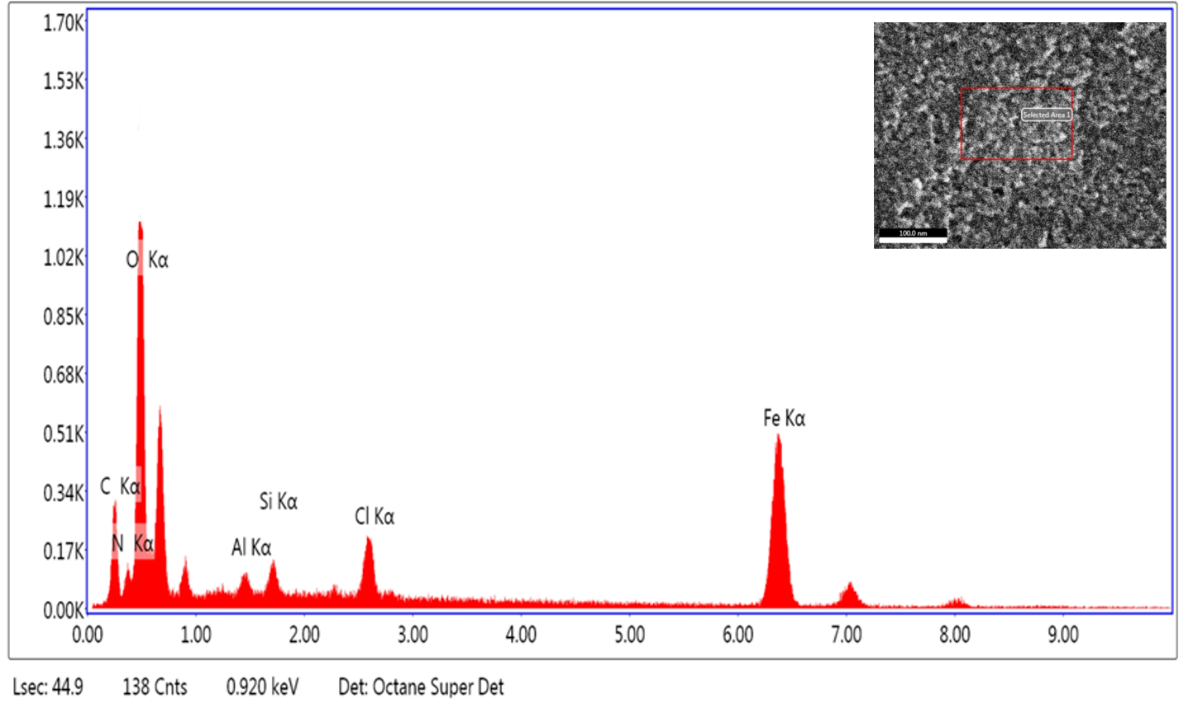


Fig. 2S. Energy-dispersive X-ray spectrum of EDT-IONPs.


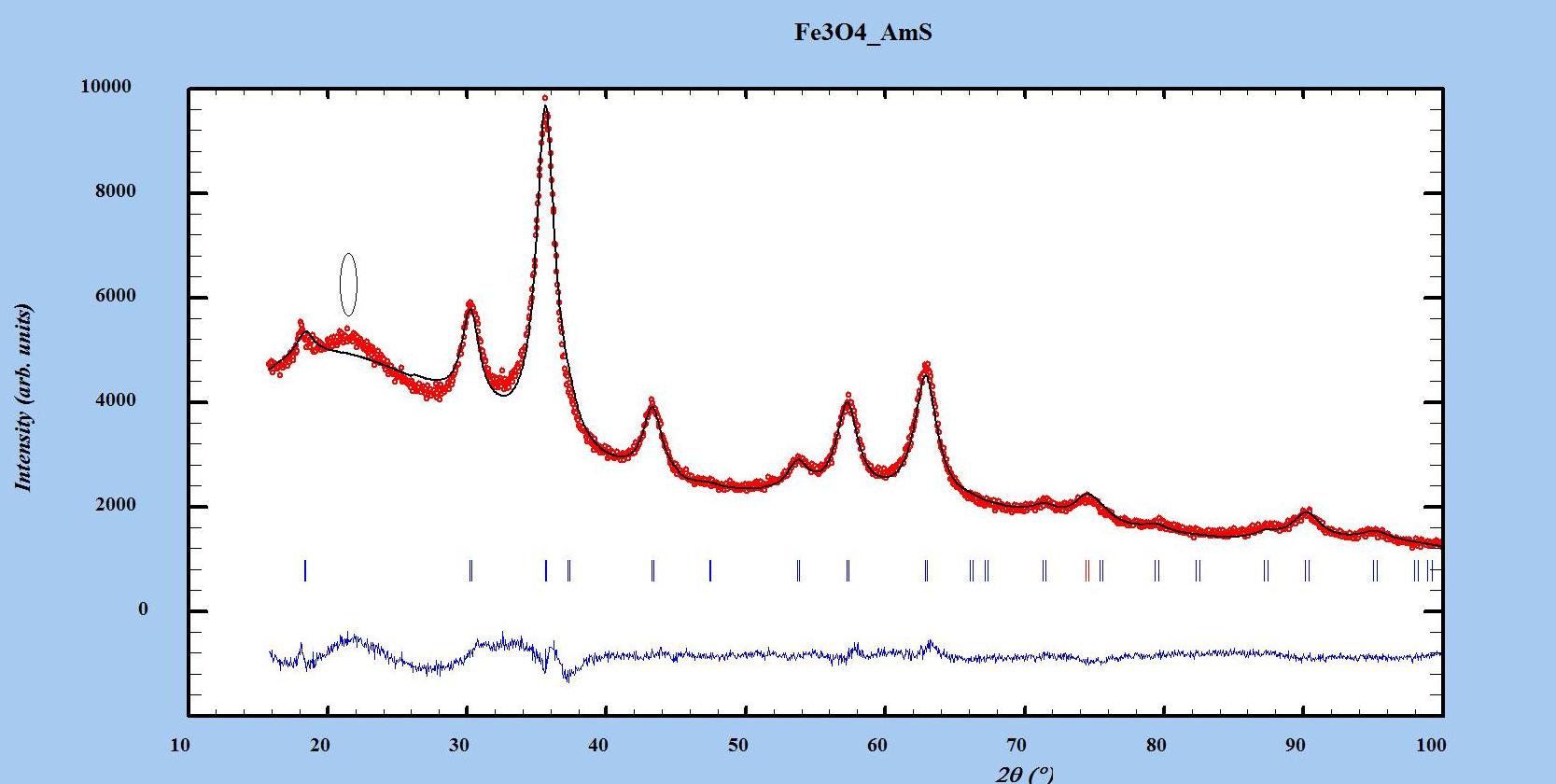


Fig. 3S. Powder X-ray diffraction pattern (red dots) and the Reitveld refinement (solid black line) of the EDT-IONP. The peaks obtained were indexed to cubic unit cell characteristic of magnetite/maghemite (Fe_3_O_4_/$\gamma-$Fe_2_O_3_) phase. A broad hump around 2 Theta 22 is from the amorphous siloxane from the EDT coating. The peaks display Scherrer broadening due to nano size.

Fig. 4S. IC50 curve of DOX on U251 cells. The cells were treated with DOX solution in cell culture media for 48 h and the MTT assay wan then conducted. The IC50 of DOX was found to be ca. 300 ng/mL


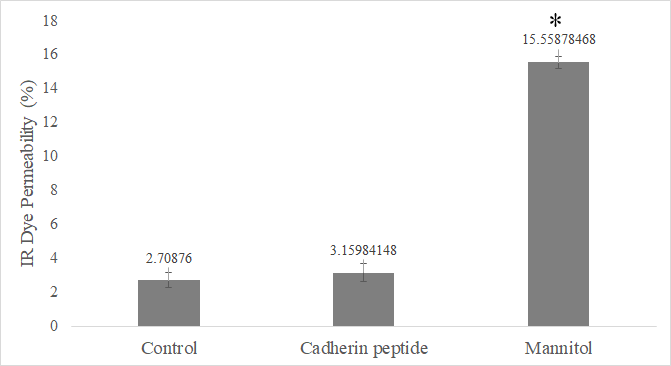


Fig. 5S. IRDye permeability across the MDCK-MDR monolayer upon 4-h treatment with peptide or mannitol within the uptake study. * indicates a significance difference compared to the control (no disrupting agent) and cadherin peptide groups (p<0.01).
